# A Web application for the management of clinical workflow in image‐guided and adaptive proton therapy for prostate cancer treatments

**DOI:** 10.1120/jacmp.v16i3.5503

**Published:** 2015-05-08

**Authors:** Daniel Yeung, Peter Boes, Meng Wei Ho, Zuofeng Li

**Affiliations:** ^1^ Department of Radiation Oncology University of Florida Health Proton Therapy Institute Jacksonville FL USA; ^2^ Ion Beam Applications Jacksonville FL USA

**Keywords:** IGRT, Web application, clinical workflow

## Abstract

Image‐guided radiotherapy (IGRT), based on radiopaque markers placed in the prostate gland, was used for proton therapy of prostate patients. Orthogonal X‐rays and the IBA Digital Image Positioning System (DIPS) were used for setup correction prior to treatment and were repeated after treatment delivery. Following a rationale for margin estimates similar to that of van Herk,^(1)^ the daily post‐treatment DIPS data were analyzed to determine if an adaptive radiotherapy plan was necessary. A Web application using ASP.NET MVC5, Entity Framework, and an SQL database was designed to automate this process. The designed features included state‐of‐the‐art Web technologies, a domain model closely matching the workflow, a database‐supporting concurrency and data mining, access to the DIPS database, secured user access and roles management, and graphing and analysis tools. The Model‐View‐Controller (MVC) paradigm allowed clean domain logic, unit testing, and extensibility. Client‐side technologies, such as jQuery, jQuery Plug‐ins, and Ajax, were adopted to achieve a rich user environment and fast response. Data models included patients, staff, treatment fields and records, correction vectors, DIPS images, and association logics. Data entry, analysis, workflow logics, and notifications were implemented. The system effectively modeled the clinical workflow and IGRT process.

PACS number: 87

## I. INTRODUCTION

Proton therapy has been used for prostate treatments for over 30 years. Published results demonstrate high rates of local and biochemical control, as well as low rates of urinary and rectal toxicities.^(2)^ Precise localization and fixation of the prostate gland, bladder, and rectum is vital during simulation, planning, and treatment. Because of the finite range and dosimetric characteristics of protons, high‐precision, fiducial‐based image‐guided radiotherapy (IGRT) is a good option for proton therapy.^3^, ^4^

In our study, four radiopaque markers (VISICOIL, IBA Dosimetry, Schwrazenbruck, Germany), one at each quadrant, were placed in the prostate gland. Two to three markers were placed in the tumor bed for patients with prostatectomies. Patients were simulated with a “comfortably filled” bladder and a distended rectum with a water‐filled rectal balloon. Computed tomography (CT) and magnetic resonance (MR) scans were acquired for treatment planning, which included two lateral or oblique fields. Digitally reconstructed radiographs (DRRs) for each field were computed and exported with the marked fiducial locations. Orthogonal X‐rays and the IBA Digital Positioning System (DIPS) were used for setup correction prior to treatment. The DIPS was run again after treatment delivery. Following a similar rationale for margin estimates,^(1)^ the daily post‐treatment DIPS data were analyzed to determine if an adaptive RT plan was necessary. We designed a Web application to automate this process and improve clinical efficiency. This improved process may help maximize the utility and potential benefits of proton therapy to offset the high capital cost in acquiring such technology.

## II. MATERIALS AND METHODS

**Figure 1:**
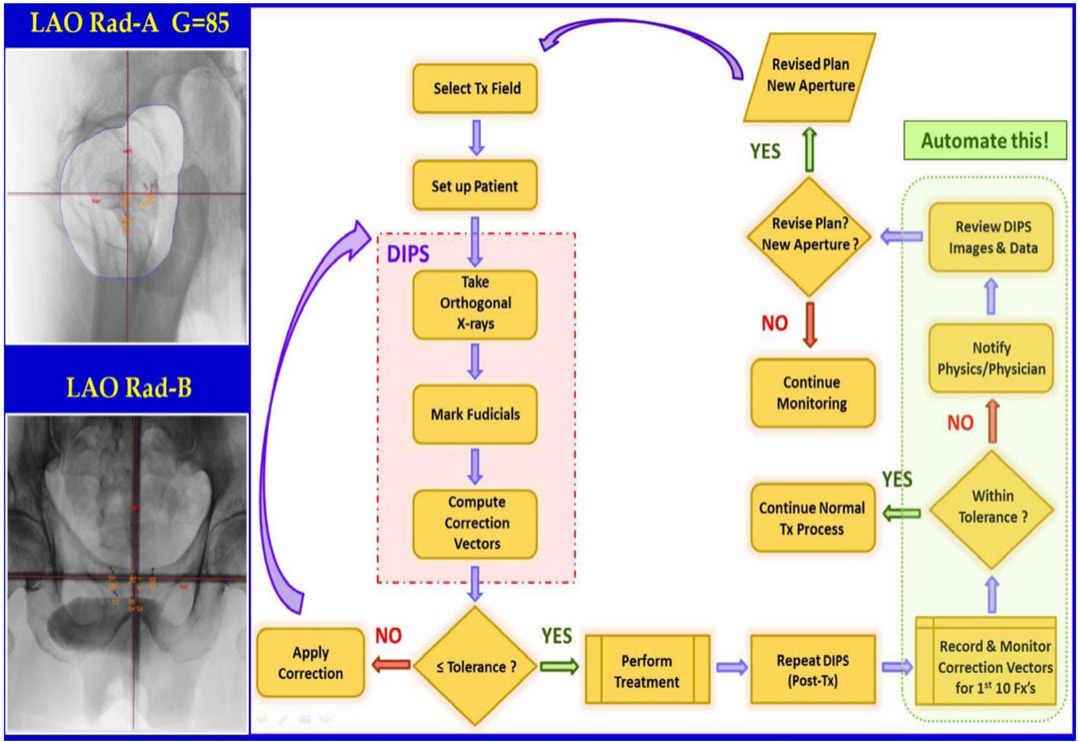
Schematic diagram of the IGRT process. Post‐treatment DIPS are taken to estimate the positional uncertainties. The e‐IGRT application aims at automating the clinical workflow for the decision process highlighted on the right.

Figure 1 outlines the IGRT process. Therapists select the treatment field and proceed with the patient setup and localization. A pair of orthogonal X‐rays are taken and the fiducials are marked. The DIPS is used to calculate and apply the required setup corrections. This process is repeated until the residual error is within tolerance. To evaluate the positional uncertainty in the treatment process, a post‐treatment DIPS is repeated. The positional errors are recorded in an Excel spreadsheet; if the values exceed the estimated uncertainty, physicians and physicists are notified. A review process then determines if revisions in the treatment plan are required to ensure adequate target coverage. There are obvious deficiencies in this clinical process as it is currently practiced. First, the entire process is manual, and related clinical data are scattered and difficult to access. The lack of central data repository also limits one's ability to perform systematic or trend analyses to help detect deficiencies or identify improvements. The manual recording of correction vectors is prone to common transcription errors. Also, the process of reporting, reviewing, and implementing corrective actions is an open loop with no proper follow‐up as a safeguard.

Thus, we created a system to automate the data analysis and decision process in the workflow to improve accuracy and clinical efficiency. A central database was used to capture vital clinical information to support concurrent access and data analysis. The carefully modeled workflow and well‐designed user interface guided the user seamlessly through the clinical process. The correction vectors were also directly retrieved from the clinical database, eliminating the possibility of transcription errors. The reporting and corrective actions were driven by the built‐in workflow, and acknowledgments were used to ensure proper follow‐up.

We also sought to address a significant flaw in the DIPS that stemmed from the “poorly designed logics” in data parsing during the import of the DICOM plan and could lead to undetected clinical errors. The DRRs for the setup and treatment fields in a given plan must be sorted in order to correctly pair the orthogonal DRRs for use in the DIPS. However, this pairing is purely based on finding pairs that have orthogonal gantry angles. When multiple fields have the same angles, the inherent logic fails and DRRs can be incorrectly paired. Subsequent use of the erroneous pair for positioning correction leads to treatment errors. However, our IGRT system provided an implementation to effortlessly traverse and navigate the clinical database. The correct association and presentation of relational data allowed the user to clearly visualize and understand the underlying relationship. Incorrect DRR pairings were easily detected. Table 1 summarizes the key design features and improvements of the Web application compared to the current manual process.

**Table 1:** Key features of the Web application designed to address the deficiencies of the current IGRT practice for prostate treatments.

*Feature*	*Current Practice*	*e‐IGRT Web Application*
Workflow	Manual	Streamlined – GUI driven. Flow execution, reporting, and acknowledgement.
		Flow control mimics optimal clinical workflow.
Clinical Data	Scattered Unorganized	Centralized SQL database.
Data Handling	Potential for transcription errors, data omissions, and misplacements	Automatic to minimize human errors.
Data Analysis	Difficult No existing tools	Data mining; trend analysis with graphic capabilities. Automatic out‐of‐tolerance detections and warnings.
Clinical Database	No access – black box Hidden errors	Full navigation. Analysis of historic data. Inconsistent data easily detectable.
Patient and System QA	Difficult No existing tools	Analysis tools support customized procedures for patient and system QA.

In the DIPS process, the tolerances for the positioning errors were 4 mm for the anterior–posterior and left–right dimensions and 6 mm for the superior–inferior dimension. These values corresponded to the planning target volume (PTV) margins chosen based on clinical observations. The post‐treatment DIPS data served as a surrogate to evaluate the intrafraction positional uncertainties. The difference in correction vectors between the post‐ and pretreatment DIPS data were calculated to determine if the respective tolerances were exceeded. The first 10 fractions were monitored with the expectation that no more than one instance (10% probability) would exceed the tolerance. After the first 10 fractions, the post‐treatment monitoring was conducted on a weekly basis. This assumption followed van Herk's margin recipe model,^(1)^ with the expectation of covering the CTV for 90% of the patients with the 95% isodose line.

**Figure 2:**
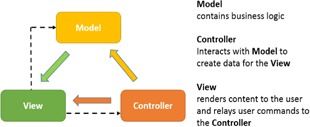
The Model‐View‐Controller architecture pattern.

**Figure 3:**
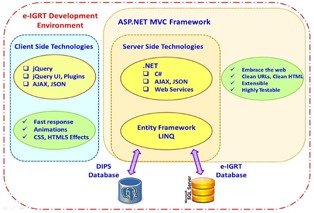
The e‐IGRT Web application development environment.

We chose the ASP.NET Model‐View‐Controller (MVC)^(5)^ framework (www.asp.net/mvc) for our Web application. The MVC architectural pattern (Fig. 2) separates an application into three main components: the model, the view, and the controller. Model objects implement the logic of the application's data domain. Views are components that display the application's user interface. Controllers handle the user interactions and bridge the data flow between the model and the views. The MVC paradigm allowed each of the three distinct layers to be developed and tested in relative isolation; combined together, they achieved a more robust and highly maintainable Web application. The MVC flow pattern is best illustrated in an example. When a user clicks a menu option related to the patient operation, the request is sent to the “Patient Controller” to invoke the requested action. If the request is for editing, the controller queries the model and database to retrieve details for the specific patient, sends them to the view for display, and awaits further user actions. Access control can be imposed at the controller level or at the individual action level within the controller. For instance, one can selectively allow certain users to view the patient information, but deny their access for the delete action. Similarly, one can restrict access of the “Account Controller” to block all underlying actions, except for the administrator user. This flexibility allows easy management and fine‐grain flow control. MVC is extensible and with lightweight HTML, client‐side technologies such as jQuery and jQuery UI could be leveraged to achieve responsive and interactive webpages. The sophisticated .NET web development environment also offers a rich set of established tools. For example, the Entity Framework was used to interface to the DIPS (Postgres) database, and a SQL Server was used for the e‐IGRT database. Figure 3 illustrates the rich e‐IGRT development environment. The design features include state‐of‐the‐art web technologies; a domain model closely matching the workflow; a database supporting concurrency and data mining; access to the DIPS database; secured user access and role management; and graphing and analysis tools. With such a robust design, the web application provides an efficient framework to manage clinical workflow.

## III. RESULTS & DISCUSSION

**Figure 4:**
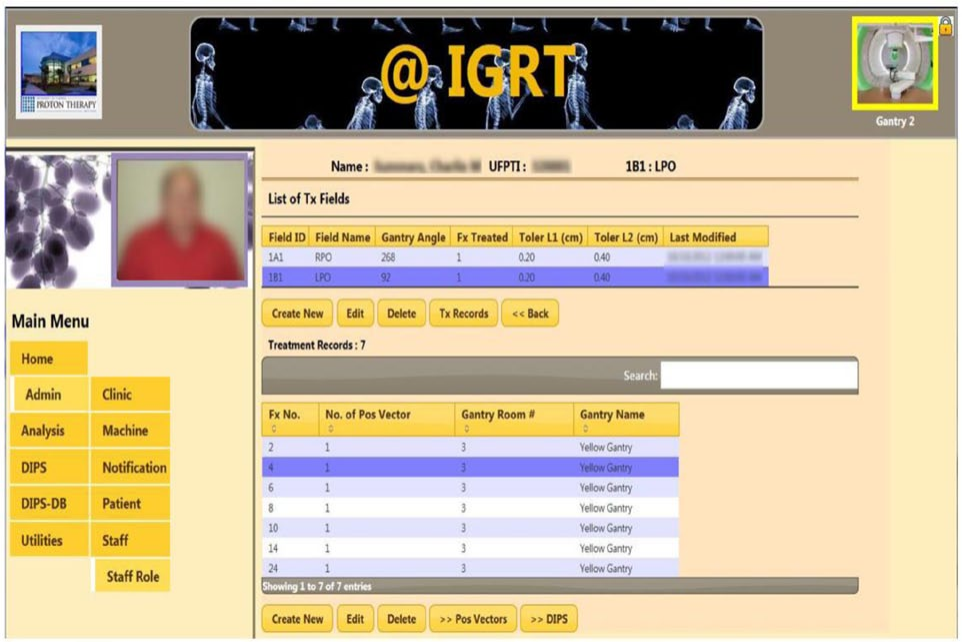
System layout of the e‐IGRT application. The design features a multilevel menu and display of patient photo for identification. User selects the gantry by clicking the icon image on the top right. A matching color border reflects the gantry room selected. The lock icon can lock or unlock the selection.

We developed a prototype using ASP.NET MVC5, Entity Framework, and the SQL database. Modeled data included patients, staff, treatment fields and records, correction vectors, DIPS images, and association logics. In this prototype, dosimetrists enter new plan information via a browser. As Fig. 4 shows, the system layout includes a multilevel menu on the left and a patient photo for identification. At the top right, an icon with color‐coding indicates the selected treatment room (gantry). The patient name and IDs are located in the center panel. This example shows the treatment fields and treatment records of the selected field. At each treatment console, the therapists in each gantry room independently select the patient and field for treatment on their respective browsers.

**Figure 5:**
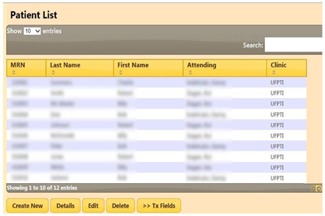
A table showing a list of patients queried from the database. Interactive selection, paging, sorting, and search are supported.

Figure 5 displays a list of patients queried from the database. Our prototype supports interactive selection, paging, sorting, and search. All these features are built‐in with the jQuery DataTable plugin. Menu options are displayed as buttons below the list. User interaction on this page invokes the patient controller and the corresponding action. For example, with a patient selected (highlighted), clicking “Edit” invokes the edit page with patient details. At the treatment consoles for each gantry, therapists can access the Web application and database concurrently, and select the patient and field scheduled for treatment.

**Figure 6:**
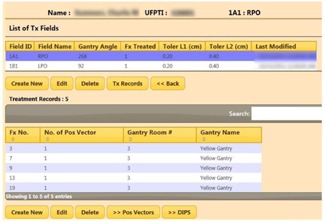
Tables showing the treatment fields of the selected patient (top) and the treatment records for the highlighted treatment field (bottom).

Figure 6 shows a table of the treatment fields and the list of treatment records for the selected field. Pre‐ and post‐treatment correction vectors are saved with the DIPS images. During the DIPS process, the correction vectors can be entered via a user interface or queried and retrieved directly from the DIPS clinical database where the vectors are captured during the localization process. The data are analyzed, and the decision logics for margin tolerance and the necessity for adaptive radiotherapy are invoked.

**Figure 7:**
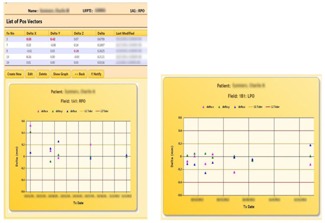
Table showing the correction vectors between the pre‐ and post‐treatment DIPS for the 1A1 field. Values exceeding the tolerances are highlighted and user is prompted to take actions. The “Show Graph” button presents a graphical analysis of the treatment records. A similar analysis for the 1B1 field is also shown.

**Figure 8:**
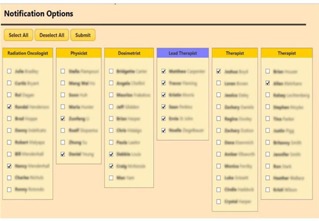
Staff lists are presented to the user for selection to send notifications. The buttons “Select All” and “Deselect All” can apply to selected (highlighted) staff group(s) for convenience. The “Submit” button will activate the “email controller” with the compiled email lists and treatment field information.

Figure 7 shows a table of the correction vectors. The vectors that exceeded the tolerances are highlighted in red. The “Show Graph” option displays the vectors in a graph with the tolerances shown as horizontal lines. The users are prompted to take appropriate actions when tolerances are exceeded. Workflow logics include notifications to physicists and physicians that are triggered, if necessary. For the latter, a “Notify” button activates a notification page where a user can select from the staff list (Fig. 8). “Submit” activates the email page with a compiled list of staff selected for notification. Notified personnel review and analyze to decide on the appropriate course of action. Events and action triggers are propagated if new plans are necessary. In this case, the workflow cycle is reiterated. Graphing and analysis tools include multimode plots and selective, comparative, and trend analyses.

**Figure 9:**
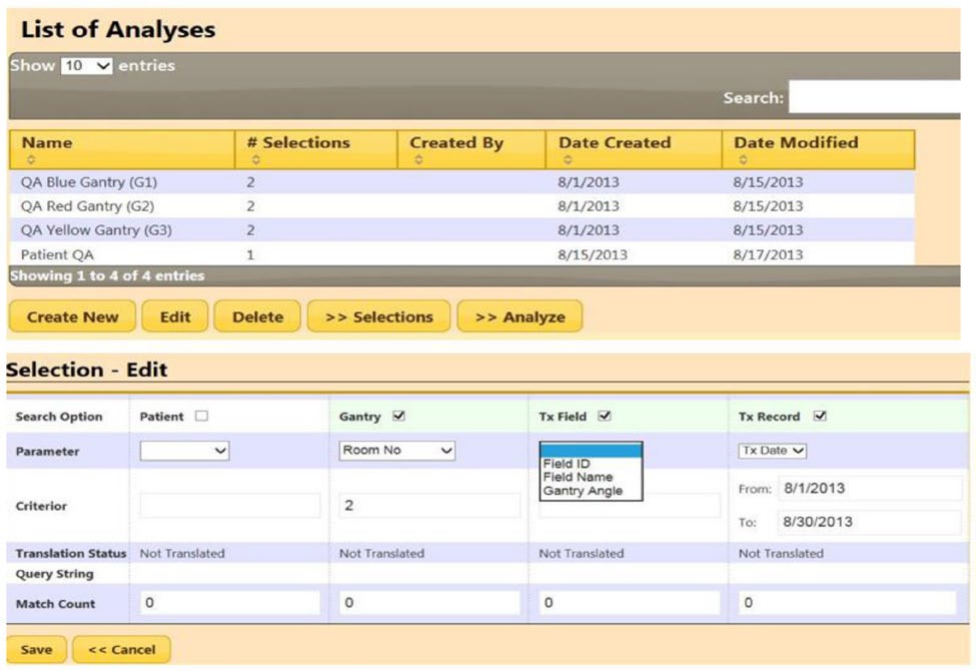
Top area of figure shows a list of analyses. Each analysis consists of a collection of selections. A selection defines what search categories and corresponding parameters to use to allow for flexible extraction and grouping of treatment records for analysis and comparative evaluations.

Figure 9 shows the analysis page. Each analysis consists of a collection of selections, including flexible options to select and group treatment records to be compared and analyzed. This tool has excellent potential for patient and system quality analysis (QA). For example, a loss of spring tension could result in an aperture shift for a gantry angle of 270° but not 90° because of gravity and the orientation of the insertion opening. A systematic displacement in aperture in DIPS could allow early detection of such a problem. A customized analysis could also scrutinize all new patient treatments to detect any anomalies that warrant further review by physicists. For example, an inexperienced therapist might produce inaccurate DIPS markings, which would surface as larger errors in the correction vectors. Our system supports navigation of the clinical DIPS database. Related information scattered across multiple tables in the relational database are correctly queried and grouped for clear presentation so that the underlying relationships can be easily comprehended.

**Figure 10:**
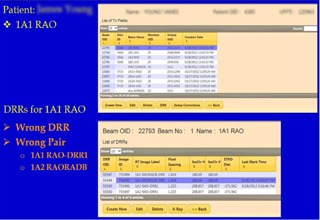
Bottom area of figures show the DRRs associated with the treatment field 1A1 RAO queried from the clinical DIPS database. An error was revealed that the system wrongly paired up the Rad B for the field 1A2 with the Rad A of 1A1 as the orthogonal pair for location.

Figure 10 shows an example of the orthogonal DRRs associated with a given field for DIPS marking and analysis. In this case, it revealed an error in the DIPS database by incorrectly associating the DRR “RadA of 1A1” (initial field) with that of “RadB of 1A2” (boost field) as the orthogonal pair for location. This error was due to a flaw in the data import and translation process of the DIPS. During plan import and data parsing, the system searched and paired up DRRs with orthogonal gantry angles as an orthogonal pair. If the gantry angles for the 1A1 and 1A2 fields were the same, incorrect pairing could occur. This type of error would be hidden from the user. If the isocenter coordinates of initial and boost fields, 1A1 and 1A2 respectively, were different, the erroneous pairing used for DIPS localization would introduce a systematic error in the calculation of the correction vector. With the ability to navigate through the clinical database and present the related entities unambiguously, the user can easily detect such mistakes. This GUI‐based navigation feature is superior to the low‐level SQL command line queries that are difficult to comprehend. This is a valuable tool for reviewing and verifying the database tables and relationships between the clinical data. In addition to prostate treatments, such errors often surface with cranial spinal irradiations where multiple adjacent spinal (PA) fields with the same gantry angles are required to cover the entire spine. The incorrectly associated spine fields require IBA engineers to manually correct the database, often leading to delays and increased clinical workloads. The navigation feature could be extended to support “write” operations, simplifying the correction of such pairing errors. The navigation, in conjunction with the analysis capability of the clinical database, will allow population‐based analysis to be performed on a vast collection of historic data. This has the potential of yielding useful information regarding optimal treatment margins. The system implementation also included security features, such as form authentication, and secured HTTP of sensitive data to ensure HIPAA compliance. The prototype is currently undergoing clinical testing and validation.

## IV. CONCLUSIONS

Our Web application manages IGRT and adaptive radiotherapy clinical workflow for prostate treatments with protons. The workflow can be effectively modeled using the ASP.NET MVC and Entity development framework. The system can be utilized to improve patient and system QA, and will likely enhance clinical quality and efficiency.
